# Epidemiology of musculoskeletal disorders among iranian adults: results from a non-communicable disease cohort study

**DOI:** 10.1186/s12891-023-06435-5

**Published:** 2023-04-22

**Authors:** Farid Najafi, Mitra Darbandi, Sepideh Kazemi Neya, Masoud Tokazebani Belasi, Neda Izadi, Yahya Pasdar, Akbar Barzegar

**Affiliations:** 1 Research Center for Environmental Determinants of Health (RCEDH), Health Institute, Kermanshah University of Medical Sciences, Kermanshah, Iran; 2 Research Centre for Prevention of cardiovascular disease, Institute of Endocrinology & Metabolism, Iran University of Medical Sciences, Tehran, Iran; 3 Research Center for Social Determinants of Health, Research Institute for Endocrine Sciences, Shahid Beheshti University of Medical Sciences, Tehran, Iran; 4 Department of Occupational Health, School of Health, Kermanshah University of Medical Sciences, Kermanshah, Iran

**Keywords:** Musculoskeletal disorders, Joint pain, Back pain, Non-communicable disease

## Abstract

**Background:**

Persistent pain and reduced mobility and dexterity are the typical characteristics of Musculoskeletal disorders (MSDs). We aimed to determine the prevalence of back and joint pain, back, and joint stiffness, arthritis, and osteoporosis among adults and their distribution according to sociodemographic characteristics and comorbidities.

**Methods:**

This cross-sectional study was conducted on 9,520 participants aged 35–65 years from baseline data of the Ravansar Non-Communicable Disease (RaNCD) cohort study, in the west of Iran. MSDs were evaluated by the RaNCD cohort study physician using a standard questionnaire. Binary logistic regression was used to determine associations and reported by odds ratios (OR) and 95% confidence intervals (CI).

**Results:**

The MSDs in women were significantly more than in men (59.06% vs. 40.94%, P < 0.001). Skeletal muscle mass (SMM) was significantly lower in subjects with MSDs, and waist circumference (WC) and visceral fat area (VFA) were higher than in the non-MSDs (P < 0.001). MSDs were significantly more common in urban men and women (OR = 1.33; 95% CI: 1.14–1.50 and OR = 1.59; 95% CI: 1.39–1.82, respectively). Obesity increased the odds of MSDs in women (OR = 1.57; 95% CI: 1.33–1.84), whereas there was no association between BMI and MSDs in men. Men with high socioeconomic status (SES) had lower odds of MSDs than men with weak SES (OR = 0.77; 95% CI: 0.64–0.92).

**Conclusion:**

The MSDs were more prevalent among older people, women, obese people and urban dwellers. Lifestyle modification, especially weight loss may be helpful in reducing and controlling MSDs.

## Background

Musculoskeletal disorders (MSDs) deal with joints, bones, muscles, and multiple body areas or systems. Persistent pain and reduced mobility and dexterity are the typical characteristics of MSDs conditions. Studies report that musculoskeletal problems are the leading cause of 15% of primary health care consultations [1]. Although MSDs are generally more prevalent among the older generation, younger individuals, especially through the peak of their income-earning years are also affected resulting in reducing their quality of life and work capacity [2, 3]. Based on data from Global Burden of Disease (GBD) 2019, around 1.71 billion individuals worldwide are suffering from MSDs, including neck pain, rheumatoid arthritis, low back pain, fractures, other injuries, amputation, and osteoarthritis. Globally, MSDs conditions account for the leading cause of disability. Low back pain, which is the most common complaint among MSDs, is the major contributor to disability in 160 countries [2–4].

A significant limitation of Mobility and dexterity caused by MSDs leads to premature retirement, decreased levels of well-being, and limited ability to attend social activities. MSDs conditions are considered the primary cause of seeking rehabilitation services around the world [5]. People living with MSDs are at a higher risk of developing other non-communicable diseases namely cardiovascular diseases and also mental health issues [4]. The number of individuals suffering from MSD conditions and related functional restrictions is rapidly growing as a result of the worldwide population aging which is extremely burdensome for both individuals and public healthcare bodies [6].

Epidemiological assessment of non-communicable diseases is among the first steps for policymakers to plan and provide appropriate and efficient healthcare resources. The current cross-sectional analysis aims to study the Epidemiology of musculoskeletal disorders among Iranian adults using data from the baseline phase of the Ravansar Non-Communicable Disease (RaNCD) cohort. The RaNCD cohort constitutes one major portion of the Iranian Adult Cohort Prospective Epidemiological Research Studies (PERSIAN). In our study, we measured MSDs including back and joint pain, back and joint stiffness, rheumatoid arthritis, and osteoporosis as outcomes.

## Method

### Study design and setting

The present study is a cross-sectional analysis of the data from the RaNCD cohort. The RaNCD cohort study is a part of the Iranian Adult Cohort Prospective Epidemiological Research Studies (PERSIAN). In the Persian cohort, all 19 cohort sites (including a representative sample of different Iranian ethnicities in different provinces) and aimed to follow up with all participants for the next 15 years [7]. More information is available at https://persiancohort.com/. Ravansar is one of the cities of Kermanshah province, which is located in the west of Iran. The baseline phase of the RaNCD study was conducted between November 2014 and February 2017, the details of which have been previously published [8]. All participants in the baseline phase of the RaNCD study entered the study (n = 10,047). Exclusion criteria included pregnant women (n = 136), participants with cancer (n = 80), participants with amputation or physical disabilities (n = 149), and missing data (n = 162). Finally, 9,520 subjects were included in the current study.

### Data Collection

#### Questionnaire information

Demographic and lifestyle information in the digital cohort questionnaire was completed face-to-face by the trained experts of the RaNCD Cohort center. The current smokers were considered to be people who smoked at least 100 cigarettes a year. According to the RaNCD study protocol, alcohol consumption was defined as drinking approximately 200 ml of beer or 45 ml of liquor once a week for at least six months. The length of time that the participant usually sleeps is 24 h. Menopause status was defined as having no menstrual period for the past 12 months.

##### Socio-economic status measurements

Socio-economic status (SES) was generated using principal component analysis (PCA) using 18 items including education level, place of residence, housing, and amenities [9]. Then the participants were ranked and classified into three groups from the lowest to the highest.

##### Physical activity measurements

The physical activity was assessed using the PERSIAN cohort questionnaire and was classified into three groups, low (24–36.5 MET/hour per day), moderate (36.6–44.4 MET/hour per day) and high (≥ 44.5 MET/hour per day) [8].

### Measurements

#### Anthropometry

Body composition components including body mass index (BMI), visceral fat area (VFA), and waist circumference (WC) were measured by Bio-Impedance Analyzer BIA (Inbody 770, Inbody Co, Seoul, Korea).

### Definitions of T2DM, CVDs, hypertension, dyslipidemia, depression, and obesity

Individuals with a systolic blood pressure (SBP) ≥ 140 mm Hg and/or diastolic blood pressure (DBP) ≥ 90 m Hg and/or those with a current use of antihypertensive drugs as hypertensive were classified as having hypertension. Fasting blood sugar (FBS) ≥ 126 mg/dL and/or treatment with antidiabetic medications were considered for the diagnosis of type 2 diabetes mellitus (T2DM). The participants are with a history of hospitalization and/or treatment for one or more heart diseases such as stroke, myocardial infarction, and coronary artery disease, and/or taking medications for cardiovascular diseases (CVDs). Dyslipidemia was defined as serum levels of total cholesterol ≥ 240 mg/dL and/or low-density cholesterol (LDL-C) ≥ 160 mg/dL and/or triglyceride (TG) ≥ 200 mg/dL and/or low-density cholesterol (HDL-C) < 40 mg/dL or taking medications for dyslipidemia [10]. Depression was defined based on the diagnosis of depression by a physician or receiving medication for depression. BMI ≥ 30 kg/m^2^ was considered obese.

### Musculoskeletal disorders

MSDs were the outcome in this study that included back pain, joint pain, back stiffness, joint stiffness, rheumatoid arthritis, and osteoporosis. According to the RaNCD cohort study protocol, back pain and joint pain were defined based on participants’ self-report of pain, and the location of pain was confirmed based on the RaNCD study cohort physician’s opinion. Pain associated with fractures, malignancies, and infections was not considered back pain. Back and joint stiffness is defined by the experience of back pain and joint pain, with morning stiffness of one hour or more. Rheumatoid arthritis and osteoporosis are based on self-reports of previous diagnoses by a specialist.

### Statistical analysis

All analysis was performed using STATA software version 14.2 (Stata Corp, College Station, Tex). Descriptive findings are reported as percentages for categorical variables as a number (percentage) and as mean ± standard deviation for continuous variables. The distribution of baseline characteristics (including demographic, anthropometry, physical activity, lifestyle, and comorbidity) variables between men and women, as well as between two groups with and without musculoskeletal disorders, was compared with the Chi-square test and t-test. Binary logistic regression analysis was performed to evaluate the association between musculoskeletal disorders and risk factors using crude and adjusted models for age, smoking, alcohol use, and sleep duration. All variables with a P-value less than 0.2 in the univariable model were included in the multivariable analysis. Odds ratios (ORs) were calculated with 95% confidence intervals (CIs) were calculated. P values < 0.05 were considered significant for all analyses.

## Results

A total of 9,520 cohort study participants with an average age of 47.27 ± 8.25 years who met the inclusion criteria for the present study were analyzed. Table 1 presents the basic information about the participants in the present study. In this study, 47.67% of the participants were men and 59.88% were rural residents. High physical activity in men was almost three times that of women (34.86% vs. 10.98%). The prevalence of CVDs, obesity, and depression was significantly higher in women than in men (P < 0.001). Furthermore, 1,334 of the studied men (29.40%) and 2,226 of the women (44.68%) had joint pain, which was significantly more in women than in men (P < 0.001). All MSDs in the studied women were significantly more than in men (P < 0.001).

**Table 1:** Baseline characteristics of studied participants, (N = 9,520)

Characteristics	Total (n = 9,520)	Men (n = 4,538)	Women (n = 4,982)	P value
	Mean ± SD or frequency (%)			
**Age (years)**	47.27 ± 8.25	46.92 ± 8.05	47.58 ± 8.41	0.001
**Residency, n (%)**				
Urban	5701 (59.88)	2811 (61.94)	2890 (58.01)	< 0.001
Rural	3819 (40.12)	1727 (38.06)	2092 (41.99)	
**Lifestyle**				
Current smoker, n (%)	1090 (11.50)	997 (22.07)	93 (1.88)	< 0.001
Alcohol use, n (%)	463 (4.86)	461 (10.16)	2 (0.04)	< 0.001
Sleep duration ( per 24 h)	7.10 ± 1.22	7.01 ± 1.19	7.18 ± 1.24	< 0.001
**Physical activity (Met h/day)**				
Low	2869 (30.14)	1576 (34.73)	1293 (25.95)	< 0.001
Moderate	4522 (47.50)	1380 (30.41)	3142 (63.07)	
High	2129 (22.36)	1582 (34.86)	547 (10.98)	
**Comorbidities, n (%)**				
Hypertension	1487 (15.62)	663 (14.61)	824 (16.54)	0.009
Diabetes	816 (8.57)	363 (8.00)	453 (9.10)	0.057
Dyslipidemia	4215 (44.28)	2413 (53.17)	1802 (36.17)	< 0.001
Cardiovascular diseases	1598 (16.79)	554 (12.21)	1044 (20.96)	< 0.001
Depression	292 (3.07)	90 (1.98)	202 (4.05)	< 0.001
**Body mass index (kg/m2)**				
Normal (18-24.9 kg/m^2^)	2860 (30.04)	1685 (37.13)	1175 (23.58)	< 0.001
Overweight (25-29.9 kg/m^2^)	41.33 (43.41)	2099 (46.35)	2034 (40.83)	
Obesity (≥ 30 kg/m^2^)	2527 (26.54)	754 (16.62)	1773 (35.59)	
**Musculoskeletal disorders**				
Back pain	2211 (23.23)	962 (21.20)	1249 (25.07)	< 0.001
Back stiffness	745 (7.83)	231 (5.09)	514 (10.32)	< 0.001
Joint pain	3560 (37.40)	1334 (29.40)	2226 (44.68)	< 0.001
Joint stiffness	472 (4.96)	124 (2.73)	348 (6.99)	< 0.001
Rheumatoid arthritis	246 (2.58)	51 (1.12)	195 (3.91)	< 0.001
Osteoporosis	242 (2.54)	31 (0.68)	211 (4.24)	< 0.001

Data are shown mean ± SD for continuous variables and n (%) categorical variables

*P- value was obtained t-test and Chi square test

MSDs were significantly higher in men than in women, and in urban residents than in rural residents (P < 0.001). High SES was 30.86% in the group with MSDs and 36.21% in the non- MSDs group (P < 0.001). Overall, the participants with MSDs had a significantly lower SES (P < 0.001). BMI, WC, and VFA were significantly higher in individuals with MSDs than in non-MSDs (P < 0.001). The SMM in the individuals with the MSDs group was significantly lower than the other group (27.01 ± 5.82 vs. 25.93 ± 5.60; P < 0.001). The prevalence of obesity was 30.17% and 23.01% in those with and without MSDs, respectively (P < 0.001). Hypertension, T2D, CVDs, and depression in individuals with MSDs were significantly higher than the non-MSDs. The use of contraceptive pills in women with MSDs was significantly higher than in women without MSDs (P < 0.001) **(**Table 2**).**

**Table 2:** Comparison of baseline characteristics of participants according to musculoskeletal condition, (N = 9,520)

Characteristics	Non- musculoskeletal disorders (n = 4,820)	Musculoskeletal disorders (n = 4,700)	P value*
	Mean ± SD or frequency (%)		
**Age (years)**	46.11 ± 8.03	48.45 ± 8.29	< 0.001
**Sex, n (%)**			
Male	2614 (54.23)	1924 (40.94)	< 0.001
Female	2206 (45.77)	2776 (59.06)	
**Residency, n (%)**			
Urban	2723 (56.49)	2978 (63.36)	< 0.001
Rural	2097 (43.51)	1722 (36.64)	
**Socio-economic status, n (%)**			
Weak	1521 (31.56)	1612 (34.31)	< 0.001
Moderate	1553 (32.23)	1636 (34.82)	
Good	1745 (36.21)	1450 (30.86)	
**Physical activity (Met h/day)**			
Low	1472 (30.54)	1397 (29.72)	0.001
Moderate	2205 (45.75)	2317 (49.30)	
High	1143 (23.71)	986 (20.98)	
**Lifestyle**			
Current smoker, n (%)	599 (12.49)	491 (10.49)	0.001
Alcohol use, n (%)	265 (5.50)	198 (4.21)	0.004
Sleep duration ( per 24 h)	7.13 ± 1.18	7.04 ± 1.26	0.002
**Anthropometry**			
Body Mass Index, kg/m^2^	27.10 ± 4.57	27.91 ± 4.60	< 0.001
Waist Circumference, cm	96.48 ± 10.36	98.10 ± 10.60	< 0.001
Skeletal Muscle Mass, kg	27.01 ± 5.82	25.93 ± 5.60	< 0.001
Visceral Fat Area, cm^2^	115.48 ± 50.10	129.05 ± 52.11	< 0.001
**Comorbidities, n (%)**			
Hypertension	618 (12.82)	869 (18.49)	< 0.001
Type 2 Diabetes	354 (7.35)	462 (9.83)	< 0.001
Dyslipidemia	2125 (44.10)	2090 (44.47)	0.708
Cardiovascular diseases	625 (12.97)	973 (20.70)	< 0.001
Depression	127 (2.63)	165 (3.51)	0.013
Obesity (≥ 30 kg/m^2^)	1109 (23.01)	1418 (30.17)	< 0.001
**Menopausal women, n (%)**	526 (23.82)	1025 (36.86)	< 0.001
**Contraceptive pills use, n (%)**	1657 (75.08)	2171 (78.21)	0.009

Data are shown to mean ± SD for continuous variables and n (%) categorical variables

*P- value was obtained t-test and Chi square test

Table 3 shows the association of demographic and anthropometric factors and comorbidities with MSDs. On the basis of multivariable logistic regression, each one-year increase in age was associated with a 1% increase in odds of MSDs in men (OR = 1.01; 95% CI: 1.01–1.02) and the corresponding value for women was 3% (OR = 1.03; 95% CI: 1.02–1.04). MSDs were significantly more common in men and women living in city (OR: 1.33; 95% CI: 1.14–1.50 and OR: 1.59; 95% CI: 1.39–1.82, respectively). Being overweight was associated with increased odds of MSDs (OR = 1.34; 95% CI: 1.15–1.56). Similarly, obesity increased the odds of MSD in women (OR = 1.57; 95% CI: 1.33–1.84), whereas there was no association between BMI and MSDs in men. Furthermore, men with high SES had lower odds of MSDs than men with weak SES (OR = 0.77; 95% CI: 0.64–0.92). In women, moderate SES was associated with a 18% increase in the odds of MSDs (OR = 1.18; 95% CI: 1.03–1.36). In addition, in men, former smokers (OR = 1.21; 95% CI: 1.01–1.47) and in women, former and passive smokers (OR: 1.90; 95% CI: 1.30–2.79 and OR: 1.13; 95% CI: 1.01–1.27, respectively) were associated with the prevalence of MSDs. CVDs were associated with a significant increase in MSDs in men (OR = 1.28; 95% CI: 1.01–1.63). However, in women, there was no significant association between comorbidities and MSDs. Menopause was associated with a 21% increase in odds of MSDs (OR = 1.21; 95% CI: 1.01–1.47).

**Table 3:** Association of demographic, anthropometric factors and comorbidities with musculoskeletal diseases using binary logistic regression

Variables	Men		Women	
	Univariable	Multivariable	Univariable	Multivariable
	Odds ratio (95% CI)		Odds ratio (95% CI)	
**Age (year)**	1.02 (1.01–1.03)	**1.01 (1.01–1.02)**	1.04 (1.03–1.04)	**1.03 (1.02–1.04)**
**Residency**				
Urban	1.17 (1.03–1.32)	**1.31 (1.14–1.50)**	1.56 (1.39–1.75)	1.59 (1.39–1.82)
Rural	Ref.	Ref.	Ref.	Ref.
**Socio-economic status**				
Poor	Ref.	Ref.	Ref.	Ref.
Moderate	0.82 (0.69–0.97)	0.84 (0.70–1.01)	1.27 (1.11–1.44)	**1.18 (1.03–1.36)**
High	0.77 (0.66–0.90)	**0.77 (0.64–0.92**)	1.08 (0.93–1.26)	1.01 (0.84–1.20)
**Physical activity (Met h/day)**				
Low	Ref.	Ref.	Ref.	Ref.
Moderate	0.98 (0.85–1.14)	1.00 (0.86–1.16)	0.93 (0.81–1.06)	1.03 (0.90–1.19)
High	1.11 (0.96–1.28)	1.11 (0.96–1.30)	0.81 (0.66–0.99)	1.05 (0.85–1.31)
**Smoke status**				
Non**-**smoker	Ref.	Ref.	Ref.	Ref.
Current smoker	1.16 (0.98–1.37)	1.16 (0.98–1.37)	1.38 (0.90–2.11)	1.19 (0.76–1.84)
Former smoker	1.36 (1.13–1.64)	**1.21 (1.01–1.47)**	2.67 (1.85–3.84)	**1.90 (1.30–2.79)**
Passive smoker	1.08 (0.93–1.25)	1.08 (0.93–1.25)	1.15 (1.02–1.29)	**1.13 (1.01–1.27)**
**BMI Level**				
Underweight	0.99 (0.65–1.50)	**-**	0.96 (0.59–1.58)	1.02 (0.61–1.70)
Normal	Ref.	Ref.	Ref.	Ref.
Overweight	1.07 (0.94–1.22)	**-**	1.37 (1.18–1.59)	**1.34 (1.15–1.56)**
Obese	1.18 (0.99–1.40)	**-**	1.69 (1.45–1.96)	**1.57 (1.33–1.84)**
**Sleep duration**				
< 6 h	1.25 (1.04–1.51)	1.20 (0.99–1.45)	1.34 (1.10–1.64)	1.18 (0.96–1.45)
6**–**7 h	Ref.	Ref.	Ref.	Ref.
≥ 8 h	0.99 (0.84–1.14)	0.98 (0.86–1.13)	0.87 (0.77–0.98)	0.94 (0.83–1.07)
**Comorbidities (yes)**				
Hypertension	1.55 (1.31–1.83)	1.17 (0.93–1.46)	1.49 (1.28–1.74)	0.95 (0.76–1.17)
Type 2 diabetes	1.14 (0.92–1.41)	**-**	1.57 (1.28–1.92)	1.13 (0.91–1.41)
Dyslipidemia	1.07 (0.95–1.20)	**-**	1.16 (1.03–1.31)	0.93 (0.82–1.05)
Cardiovascular diseases	1.61 (1.35–1.93)	**1.28 (1.01–1.63)**	1.64 (1.42–1.89)	1.20 (0.98–1.47)
Depression	0.94 (0.62–1.44)	**-**	1.39 (1.04–1.86)	1.25 (0.92–1.71)
**Menopausal women (yes)**	**-**	**-**	1.86 (1.65–2.11)	**1.21 (1.01–1.47)**

## Discussion

The current cross-sectional study provides thoughtful insight into the epidemiology of MSDs among Iranian adults. Based on this study, several factors, including aging, living in urban areas, obesity, socioeconomic status, and comorbidities are significantly associated with MSDs.

A meaningful association was found between aging and MSDs in both men and women studied groups. Residents of urban areas were revealed to suffer far more MSDs compared to the ones living in rural areas. In addition, our results showed women were shown to be more among the population with MSDs. (59.06% vs. 45.77%). A similar study showed that chronic lumbar and cervical back pain were more prevalent in the women compared to the studied men [11]. Generally, previous studies have shown that female gender is a risk factor for MSDs [12–14]. However, scientific evidence shows men are more likely to adopt hazardous behaviors, such as the use of narcotics to alleviate the suffering caused by chronic pain [15] Vega-Fernández et al.‘s study also showed that MSDs were more in urban areas than in rural areas [14]. Therefore, the association between demographic factors and MSDs was consistent with previous studies.

Regarding SES, MSDs were found significantly less common in men who were in a high compared to those with weak SES. In contradiction, MSDs among women within the moderate SES population were shown to be remarkably more prevalent. In the current study, generally, the participants with MSDs were found to have a significantly lower socio-economic status (P < 0.001). This result is similar to the finding of the study on the Australian population clarifying that MSDs, including arthritis, chronic back pain, gout, osteoporosis, spondyloarthropathies, rheumatoid arthritis, and the chronic associated pain was more prevalent among those individuals of lower socioeconomic status [16]. Another study also revealed that residents of socially deprived regions experienced more musculoskeletal symptoms [1]. It is concluded that SES is an important factor in the occurrence of MSDs.

In the present study, while obesity and being overweight were significantly related to MSDs in women individuals, a similar association was not observed among the studied men population. A hypothesis would be inactivity caused by pain, which is probably more prevalent in women and the following overweight. In a longitudinal epidemiological study, a strong association was shown between lumbar back pain and BMI > 30 [11]. Obesity accounts for a wide range of MSDs, as well as serious disabilities, and reduced quality of life [17–19]. Several data regarding the positive association between obesity and chronic low back pain are available [20, 21]. In these studies, adiposity was mainly determined based on BMI. However, information on low back pain may not be conveyed sufficiently by BMI, as it is a composition of several factors like fat and lean mass. In our study, not only BMI but also WC and VFA were measured and analyzed, resulting in a more precise estimation of the body fat distribution. A cross-sectional study revealed that people who suffer from chronic back pain tend to have a higher proportion of their body fat located in their limbs compared to people without chronic low back pain [22]. However, obesity affects people’s lifestyle, especially reducing physical activity, and can indirectly lead to MSDs. On the other hand, there is a two-way relationship between obesity and MSDs, and people with skeletal and joint pain have less physical activity, which helps to gain weight [23, 24]. Therefore, longitudinal studies are recommended for a better understanding of the relationship between MSDs and obesity.

The odds of developing hypertension, diabetes, and dyslipidemia were higher in people with MSDs in both sexes, although it was not statistically significant. Similarly, a study has shown that the prevalence of T2DM in men with and without back pain is not significantly different from each other [25]. Although the associations were not significant, but the association between metabolic diseases and MSDs, some factors such as physical activity and obesity may play a mediating role. However, our findings showed, CVDs were associated with a significant increase in MSDs in men. This is in line with a longitudinal study in adults that showed a strong association between lumbar back pain and CVDs (OR: 4.58) and introduced obesity as a mediator of the association between back pain and CVDs [11].

The use of contraceptive pills in women with MSDs was shown to be notably higher compared to the women without MSDs (P < 0.001). The number of menopausal women was also higher among the population with MSDs. However, menopause was associated with a 21% increase in odds of MSDs. Studies show an increase in back pain when women enter menopause, and it has been observed that low back pain is more common in postmenopausal women than in men of the same age [26–28]. Sex steroids have positive effects on musculoskeletal health. Estrogen plays an important role in the skeleton of body and the connective tissue system in general, and estrogen deficiency around menopause negatively affects the health of bones, muscles, collagen, tendons, ligaments, cartilage, synovial membrane, and joint capsule [29].

## Limitations

The strength of the present study was the use of data from a large population. The current study faced several limitations. Firstly, due to the cross-sectional nature of the present study, a longitudinal study is required to confirm the results. Secondly, the degree and severity of pain were not measured which in itself restricted the possibility to interpret the findings. Moreover, outcome evaluation was conducted based on participants’ self-reporting followed by RaNCD study cohort physician confirmation with no para-clinical data available which can interfere with the results. On top of that, the findings of the present study cannot be generalized to other populations due to some substantial reasons. The first and most important reason is that the studied population and especially the studied women were super active individuals doing demanding careers namely farming and Livestock farming which were far more physically active compared to other counterpart populations. Consequently, further investigations after mitigating the limitations of the current study would be strongly recommended.

## Conclusion

The findings of this study showed that MSDs are more common in women and older people. MSDs were significantly less common in men and women living in rural areas. Obesity, High and moderate SES increased the odds of MSD in women. Lifestyle modification, especially weight loss and increased physical activity may be helpful in reducing and controlling MSDs.

## Data Availability

All data generated and analyzed during this study are included in the manuscript.

## Abbreviations

BMI: Body mass index
HDL-C: High-density lipoprotein cholesterol
LDL-C: Low-density lipoprotein cholesterol
TG: Triglycerides
T-C: Total cholesterol
FBS: Fasting blood sugar
MSDs: Musculoskeletal disorders
SMM: Skeletal muscle mass
GBD: Global Burden of Disease
RaNCD: Ravansar Non Communicable Disease
*OCP*: Oral contraceptive pills
SES: Socio-economic status
PERSIAN: Prospective Epidemiological Research Studies in IrAN
WHR: Waist hip ratio
WC: Waist circumference
VFA: Visceral fat area
PCA: Principal component analysis
T2DM: Type 2 diabetes mellitus
CVDs: cardiovascular diseases
OR: Odds ratio
CI: Confidence interval
